# The Importance of Crosslinking in Electrospun Membranes for Water Contaminant Removal

**DOI:** 10.3390/polym17070988

**Published:** 2025-04-05

**Authors:** Peio Martinez-Goikoetxea, José Manuel Laza, Julia Sanchez-Bodon, José Luis Vilas-Vilela, Antonio Veloso-Fernández

**Affiliations:** 1 Grupo de Química Macromolecular (iMacroMat), Departamento de Química Física, Facultad de Ciencia y Tecnología, Universidad del País Vasco UPV/EHU, 48940 Leioa, Spain; pmartinez066@ikasle.ehu.eus (P.M.-G.); josemanuel.laza@ehu.eus (J.M.L.); julia.sanchez@ehu.eus (J.S.-B.); joseluis.vilas@ehu.eus (J.L.V.-V.); 2 BCMaterials, Basque Center for Materials, Applications and Nanostructures, UPV/EHU Science Park, 48940 Leioa, Spain

**Keywords:** water remediation, biopolymer membranes, electrospinning, chitosan, alginate, poly vinyl alcohol, biocharcoal, crosslinking, methacrylation, methylene blue

## Abstract

Traditional water purification systems often rely on synthetic materials that pose environmental risks due to their non-biodegradability and the potential release of harmful substances. To address these concerns, natural polymer-based membranes are being developed as a sustainable and environmentally friendly alternative for water treatment due to their biodegradability, low toxicity, and chemical versatility. These materials are particularly suitable for removing a wide range of contaminants due to their high selectivity and water permeability. Despite their benefits, challenges such as improving mechanical strength, durability, and resistance to fouling persist. Ongoing research continues to optimize the performance of electrospun membranes to meet modern water treatment demands. For this purpose, crosslinking via thermal initiators azobisisobutyronitrile (AIBN) and 2,2’-azobis(2-amidinopropane)dihydrochloride (V50) and chemical crosslinking by glutaraldehyde (GA) vapor have been studied for methacrylated chitosan and alginate. In addition, biocharcoal has been introduced into the membranes to enhance their functional properties. The development of natural polymer-based membranes combined with biocharcoal presents a promising and scalable solution for sustainable water purification, playing a crucial role in reducing pollution and preserving vital water resources for future generations. In this study, we demonstrate that the crosslinking effect plays a key role in maintaining the stability of alginate-based membranes in an aqueous environment while enhancing their adsorption capacity for methylene blue dye, making them promising for water purification applications.

## 1. Introduction

The demand for clean water is one of the most pressing global challenges today, driven by increasing pollution and the depletion of freshwater resources due to increasing industrial and agricultural activities [1]. Traditional water purification technologies often rely on synthetic materials that, while effective, come with significant environmental costs due to their non-biodegradability and the potential release of hazardous substances [2]. In response to these concerns, the development of membranes from natural polymers has gained considerable attention as a sustainable alternative for water purification [3]. Natural polymers, derived from renewable sources such as cellulose, chitosan, and alginate, offer a range of benefits, including biodegradability, low toxicity, and the ability to be tailored for specific filtration needs [4,5,6].

Natural polymer-based membranes are particularly well suited for addressing the issue of water contamination, which involves a diverse array of pollutants, including heavy metals, organic compounds, and microorganisms [7]. These membranes can be designed to offer high selectivity, efficiently removing contaminants while maintaining optimal water flow. The inherent properties of natural polymers, such as their hydrophilicity, biocompatibility, and ability to form stable and functional structures, make them ideal for membrane fabrication. Additionally, the potential for functionalizing these materials with specific additives or coatings further enhances their filtration efficiency and lifespan [8].

In contrast to synthetic polymer membranes, which often require energy-intensive manufacturing processes and generate non-degradable waste, natural polymer membranes provide an environmentally friendly alternative. Their biodegradability ensures that, at the end of their life cycle, they can decompose without leaving harmful residues in the ecosystem [9]. This aligns with the growing global emphasis on reducing the ecological footprint of industrial processes and products for the sustainable development of technology and its applications. Moreover, natural polymers are abundant and cost-effective, making them an attractive option for large-scale applications, particularly in regions where access to clean water is limited. 

However, the successful implementation of natural polymer membranes in water purification is not without challenges. Issues such as mechanical strength, durability under varying water conditions, and resistance to fouling must be addressed to ensure their long-term viability. These aspects reduce the lifetime of the membranes, creating an upkeep cost both for repairing and cleaning the product to avoid bacterial and algal growth that diminishes water flow and contaminant uptake efficiency [10]. Ongoing research is focused on optimizing the performance of these membranes, with advances in materials science offering new insights into how natural polymers can be engineered to meet the demands of modern water treatment systems [4].

As the need for sustainable water purification technologies grows, the development of membranes from natural polymers represents a promising approach to achieving clean water while minimizing environmental impact [11]. The potential to create efficient, scalable, and eco-friendly filtration systems highlights the importance of continued innovation in this field, with the goal of reducing pollution and preserving water resources for future generations. In this context, there are several methods for membrane synthesis that have been developed: namely, phase inversion and electrospinning are some membrane production processes employed with biopolymers, each taking advantage of and optimizing a specific characteristic of the membrane or its material. In the case of phase inversion, the membrane is produced by controlled precipitation, which offers a wide array of biopolymers and solvents to work with while giving easy access to size manipulation [12,13]. In the case of electrospinning, it uses electrostatic repulsions to create thin solution jets, which help evaporate the solvent and let polymeric chains solidify in randomly ordered nanofibers [14,15]. This method allows for creating structures with high available surface per unit volume, enhancing the interactions provided by the material employed and improving adsorption capabilities [16]. Something to consider when using this technique for water remediation is its limited rate of production (normally, in the scale of a few to tens of milligrams per hour), a challenge that needs to be overcome to become a suitable option for the thousands of liters per hour scale of wastewater created in textile industries. Certain paths in development to enhance production are multi-jet and nozzle-free electrospinning, both of which create several solution flows to accelerate the creation of a high-mass membrane [17,18].

The aim of this research is to develop membranes based on the biopolymers chitosan (Chi) and alginate (Alg) using the electrospinning technique. These biopolymers were modified and blended with polyvinyl alcohol (PVA), with the objective of easing fiber formation in a way similar to other procedures [19,20]. The biopolymers were modified through methacrylation, enabling thermal crosslinking via initiators and chemical crosslinking using glutaraldehyde (GA), offering a new path along photo-crosslinking [21] to the well-established ionic [22] and interpolymeric [23] crosslinking. The crosslinking approach was employed to improve the physicochemical properties of the membranes and reduce their solubility in water; however, this brings out the need to search for environmentally friendly products to avoid further pollution in aqueous media. Finally, biocharcoal was incorporated into the system as a filler to further increase the membranes’ adsorption capacity. The resulting membranes aim to enhance the adsorption of pollutants found in water. For this purpose, adsorption and desorption of methylene blue (MB) contaminants has been studied to determine the possibility of reusing the membranes.

## 2. Materials and Methods

### 2.1. Reagents

As biopolymers, 50–190 kDa molecular weight chitosan (Chi) with 75–85% deacetylation degree (DD) and 30–100 kDa molecular weight sodium alginate (Alg) derived from *Brown algae* with 65:35 G:M ratio (viscosity of 4–12 cP at 1% concentration in water) were used (both from Sigma Aldrich, St. Louis, MO, USA). Methacrylation reaction of both biopolymers was performed using methacrylic anhydride (99% purity, Sigma-Aldrich, St. Louis, MO, USA). Sodium hydroxide (NaOH, 98% purity, PanReac, Darmstadt, Germany) was employed to adjust the pH. In order to perform crosslinking in the membrane thermal initiators azobisisobutyronitrile (AIBN, Merck, Darmstadt, Germany) and 2,2’-azobis(2-amidinopropane)dihydrochloride (V50 Wako, Osaka, Japan) were used, as well as 25% glutaraldehyde water solution vapor (GA, PanReac). To prepare the electrospinning mixture solutions, 83–124 kDa molecular weight polyvinyl alcohol (PVA) with 88% hydrolysis degree was utilized (Sigma-Aldrich) in combination with biopolymers in 8:2 proportion using 8% total polymer mass. For biocharcoal synthesis, anhydrous glucose with 96% purity (Sigma-Aldrich) was used.

### 2.2. Methacrylation of Biopolymers and Crosslinking

To obtain methacrylated chitosan (MChi) and methacrylated alginate (MAlg), 1.5 g of each biopolymer was dissolved in 100 mL of different solvents: 1% acetic acid solution for Chi and pure Millie Q water for Alg. Methacrylic anhydride was added upon obtaining 1.5% *w*/*v* (mass solute/volume solvent) after stirring for 3 days. In the case of Chi, 6 mL was directly added, while in the case of Alg, 22 mL of methacrylic anhydride was gradually added dropwise over 2 h, maintaining the pH between 8 and 11 by adding 5 M NaOH solution dropwise as necessary. Since methacrylic anhydride is insoluble in water, the dropwise addition allowed for controlled reaction conditions, preventing rapid acidification or alkalinization, which could lead to Alg degradation [24]. The Chi mixture was allowed to react at 40 °C for 24 h to achieve a high degree of methacrylation. In the case of Alg, once the anhydride addition was complete and the pH was balanced at 8, the reaction was allowed to proceed at 5 °C for 24 h [24]. Both reaction products were dispersed in 75 mL of water and subjected to dialysis using cellulose membranes (12–14 kDa for Chi and 3.5 kDa for Alg) to remove unreacted methacrylic anhydride and methacrylic acid formed during the reaction. Finally, the purified polymers were lyophilized at −50 °C under 0.1 atmospheres vacuum.

**Scheme 1:**
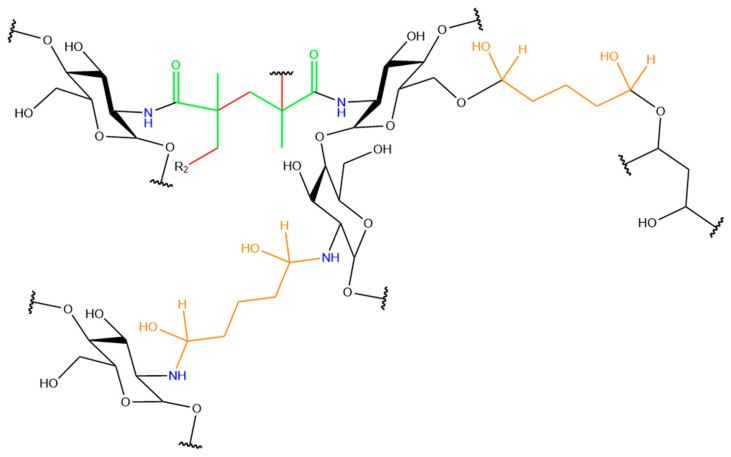
An example of crosslinking of PVA:MChi sample: in green is the methacrylate group; in red are bonds created by V50 crosslinking between methacrylate double bonds; in yellow, crosslinking from GA.

The methacrylation was performed in order to crosslink methacrylate groups in the fibers structure as can be observed in Scheme 1. This process was facilitated by thermal initiators AIBN (dissolved in methanol) and V50 (dissolved in water), also using a 25% glutaraldehyde water solutions vapor [25]. 

### 2.3. Synthesis of Biocharcoal 

A 25% glucose solution was prepared in Millie Q water for the synthesis of biocharcoal. This solution was then placed in a 50 mL steel reactor and heated to 220 °C at a rate of 10 °C·min^−1^. After maintaining this temperature for 4 h, the reactor was cooled in a water bath. Once cooled, the excess liquid was removed by decantation, and the solid product was dried at 100 °C for 24 h [26]. In order to obtain a homogeneous biochar powder, a cryogenic mill 6770 Freezer/Mill SPEX SamplePrep (Metuchen, NJ, USA) was used. The samples were first exposed to liquid nitrogen for 5 min, followed by ten grinding cycles under −195.8 °C cryogenic conditions for 2 min each cycle at a speed of 15 cps. The processed product was characterized using SEM analysis.

### 2.4. Proton Nuclear Magnetic Resonance (^1^H-NMR)

Methacrylation degree (MD%) of Chi and Alg was determined using ^1^H-NMR spectroscopy. Specifically, ^1^H-NMR spectra were performed in D_2_O acidified with acetic acid-d_4_ at a concentration of 1% *v*/*v*. Measurements were conducted using a Bruker Avance 500 MHz spectrometer (Rheinstetten, Germany) at 25 °C, with a polymer concentration of 1.1% *w*/*w*.

For chitosan, as described in the literature [27], MD% was calculated by analyzing the ratio of the integral areas (I) of H_2_–H_6_ proton signals (2.8–4.3 ppm) from the N-acetyl-glucosamine units and the protons (H_a_ and H_b_) from themethacrylate units at 5.6 and 5.8 ppm, respectively (Equation (1)).

(1) $$\%{DM}_{Chi}=\frac{\frac{{(I}_{H_{a}}+I_{H_{b}})}{2}}{\frac{{(I}_{H_{2}}+I_{H_{3}}\ldots +I_{H_{6}})}{6}}\times 100$$

In the case of Alg, based on the literature and the assumption that only guluronic acid (G) units undergo methacrylation, the guluronic acid content (G%) and degree of methacrylation (MD%) were determined using Equations (2) and (3), respectively. Here, H_G_ and H_M_ represent the integrals of the hydrogen signals (I) from the anomeric carbons of guluronic (5.1 ppm, H_G_) and mannuronic (4.7 ppm, H_M_) units. As in Chi, H_a_ and H_b_ correspond to the integrals of the two olefinic protons of the methacrylic group, appearing at 5.8 ppm and 6.2 ppm, respectively [28].

(2) $$\%G=\frac{I_{H_{G}}}{I_{H_{G}}+I_{H_{M}}}\times 100$$

(3) $$\%{DM}_{Alg}=\%G\times \frac{\frac{{(I}_{H_{a}}+I_{H_{b}})}{2}}{I_{H_{G}}}\times 100$$

### 2.5. Viscosity Measurement

A programmable Brookfield DV2TRV rotational viscometer (Middleboro, MA, USA) was employed to measure viscosity within the range of 100 to 40 million centipoise (cP). This instrument is capable of measuring density at various temperatures as well. It is equipped with multiple spindles to accommodate different precision and speed requirements. In this study, the SC4-21 spindle for low-volume samples was utilized. 

### 2.6. Electrospinning Process

For all samples, the polymer solution was placed in a 5 mL Terumo plastic syringe fitted with a steel needle. Electrospinning was conducted under different conditions. The Results section presents the complete optimization process of the electrospinning conditions, detailing the key parameters adjusted to achieve a uniform and stable fiber formation. For the PVA:MChi samples, the applied voltage with a HiTek Power OL400/303PD (HiTek Power Ltd., West Sussex, UK) power supply was 20 kV; using a syringe pump (Syringepump NE-300, New Era Pump Systems Inc., Farmingdale, NY, USA), a constant polymer solution flow rate of 0.35 mL h^−1^ was provided through a syringe needle of gauge 20 G. The fibers were collected on a homemade collecting plate 16 cm away from the needle. Meanwhile, the PVA:MAlg samples were prepared using 18 kV voltage, 0.2 mL h^−1^ flow rate, 18 G needle gauge, and a needle-to-collector distance of 14 cm. For all samples, temperature was kept between 18 and 21 °C, and ambient humidity values were kept between 48 and 52%. 

In terms of solvent, PVA:MAlg samples were dissolved exclusively in Millie Q water, while PVA:MChi samples required a 1 M acetic acid solution. The volume of solution in the syringe was typically between 1 and 2 mL with the system operating for 30 min to perform visual inspections and obtain SEM images (using 0.17 mL or 0.1 mL depending on the setup). However, for membrane extraction, a minimum runtime of 60 min was preferable (using 0.35 mL or 0.2 mL according to the system) as it produced thicker membranes with prolonged processing times. After 24 h of room temperature drying, the fiber mats were detached from the collectors for characterization.

### 2.7. Scanning Electron Microscopy (SEM)

Scanning electron microscopy (SEM) was employed to capture images of the fibers obtained through electrospinning utilizing the HITACHI S-4800 model (Tokyo, Japan). To enhance the resolution of the samples, it was necessary to increase their electrical conductivity by coating them with a nanometric gold layer using the EMITECH K550X (Quorum Technologies Ltd., Laughton, East Sussex, UK) tool. Finally, the average diameter and distribution of the fibers were determined using ImageJ Version 1.54p software [29]. This technique was also employed to determine the crosslinking effect and water immersion effect in the fiber morphology.

### 2.8. Differential Scanning Calorimetry (DSC)

Thermal transitions were studied in a TA Instruments DSC25 instrument (New Castle, DE, USA). Samples, 7–10 mg in aluminum pans, were subjected to several heating and cooling scans from −20 °C to 260 °C at 10 °C·min^−1^ under nitrogen flux of 20 mL·min^−1^.

### 2.9. Dynamic Mechanical Analysis (DMA)

Membranes’ mechanical properties were evaluated in a DMA1–METTLER TOLEDO instrument (Greifensee, Switzerland) equipped with STAR© v14.0 software for curve analysis set in tensile mode. The samples (1 mm × 5 mm × 0.02 mm) were subjected to a heating process from −20 °C to 250 °C at a rate of 3 °C min^−1^ while being deformed at a frequency of 1 Hz with a displacement of 20 µm. The deformation was analyzed within the linear viscoelastic range (LVR) to determine the storage modulus (E′), representing the elastic response, and the loss modulus (E′′), representing the viscous response. The ratio of these two parameters, known as the loss factor (tan δ = E′′/E′), was used to identify the glass transition temperature (T_g_), which corresponds to the maximum point on the tan δ curve.

### 2.10. Thermogravimetric Analysis (TGA)

TGA analysis was performed using an SDT Q600 thermal gravimetric analyzer (TA instruments, New Castle, DE, USA). Samples, 10–20 mg, were heated at 10 °C·min^−1^ from 50–500 °C under a nitrogen flow (20 mL·min^−1^). The initial thermal degradation temperature (T_i_) was set at 2% of weight loss from the first derivative (DTG), and the maximum degradation temperatures of the membranes (T_dx_) were evaluated. 

### 2.11. Methylene Blue (MB) Adsorption and Desorption

In order to test the use of these films as dye adsorbents in water purification applications, an aqueous solution of MB (9 mg/L) was prepared as a model of wastewater. This choice was driven by its extended use in the textile industry due to its high adhesion to cotton fibers [30], producing high amounts of dye-polluted wastewater that pose a threat to aquatic and human life [31]. Given the cationic nature of this dye, it is expected to show adsorbance in Alg membranes [32], which could help reduce this dye’s effect on the environment. Samples of different self-standing membranes with a surface area of 1.5 ± 0.5 cm^2^ and weight of 2 ± 1 mg were introduced in vials containing 4 mL of the prepared MB solution. After adsorption time, the remaining dye in each solution was quantified by UV–VIS spectroscopy. Each solution absorbance at 663 nm was monitored periodically (0.5, 1, 2, 2.5, 3, 4, 5, and 6 h) on a Cintra 303 UV–Visible Spectrometer (GBC Scientific Equipment Ltd., Melbourne, Australia), employing a corresponding standard curve for MB dissolved in Millie Q water.

In order to verify the possibility of reutilization, the extraction of MB was followed over a period of 2 h via UV–Vis spectroscopy by immersing the membranes in 4 ml of HCl aqueous solution of pH 2. 

## 3. Results

The chemical modification of pristine Chi and Alg was achieved through a single-step reaction with methacrylic anhydride. In this context, the primary amine groups (−NH_2_) of Chi or the hydroxyl groups (−OH) of Alg undergo a nucleophilic attack on the carbonyl group of methacrylic anhydride, resulting in the formation of a new amide or ester bond, respectively. To confirm and quantify the incorporation of methacrylic groups into the polysaccharides, ^1^H-NMR analyses were conducted. The ^1^H-NMR spectra used to determine the degree of methacrylation (MD%) are displayed in Figures S1 and S2 (see Supporting Information). Signals that are useful for quantification are clearly visible, enabling the use of Equations (1) and (2). In the ¹H-NMR spectrum of MChi (Figure S1), the methyl protons of the acetyl group of chitosan appeared as a singlet at 2.0 ppm, while glucosamine ring protons (H_2_–H_6_) were observed between 2.8 and 4.3 ppm. Additional peaks at 5.6 and 5.8 ppm corresponding to the introduced olefinic protons (H_a_ and H_b_, respectively) and a distinct signal at 1.85 ppm attributed to the methyl (−CH₃) protons of the methacrylate moiety confirmed the success of the methacrylation reaction. In the ¹H-NMR spectrum of MAlg (Figure S2), two anomeric protons of guluronic and mannuronic acid units were observed at 5.1 and 4.7 ppm, respectively. Similar to MChi, two signals at 5.8 and 6.2 ppm (H_a_ and H_b_, respectively) corresponding to the olefinic protons and the methyl protons at 1.95 ppm of the methacrylate units were observed, corroborating again the success in the metacrylation reaction. Additionally, the degree of methacrylation (MD%) of Chi and Alg, as well as the guluronic acid content (G%) in the Alg chain, were calculated using Equations (1)–(3). The MChi sample exhibited a consistent MD% of 21%, whereas MAlg showed a significantly higher MD% of 40%, with a G% of 88%. This increase was attributed to the larger amount of methacrylic anhydride used during the methacrylation of Alg.

### 3.1. Electrospinning of Chitosan-Based Fibers

For the electrospinning process, different system parameters were optimized to ensure successful fiber formation. Voltage serves as a force counteracting viscosity, facilitating the formation of the Taylor cone and polymer jetting by modulating the applied electric field. Adjusting the flow rate controls the volume of solution passing through the syringe, influencing interactions within the Taylor cone and determining the polymer content in the electrospun jet. Increasing the distance between the syringe and collector enhances solvent evaporation, allowing the polymer fibers to solidify within the desired range. Additionally, variations in syringe size impact the flow rate, thereby influencing the amount of solution present in the Taylor cone [33].

Initially, electrospinning attempts using both unmodified and methacrylated biopolymers failed to produce fiber formation (spherical particles of 0.4 ± 0.1 µm diameter). Consequently, PVA:biopolymer blend was introduced to enhance the electrospinning process and achieve successful fiber formation [34]. The absence of fiber formation can be attributed to repulsive forces [35]. Since Chi needed to be dissolved in an acidic environment, it formed a polycation, leading to repulsive interactions between the positive charges on the chains. These repulsive forces were stronger than the attractive forces of hydrogen bonding between hydroxyl groups, preventing the formation of fibers. Additionally, viscosity played a crucial role. In fact, unmodified Chi with high viscosity (5223 ± 612 cP) complicated the formation of a stable Taylor cone, hindering the homogeneous stretching required for fiber formation. In contrast, MChi presented a much lower viscosity (555 ± 27 cP). Despite achieving a semi-stable Taylor cone, the strength of the negative interactions was too high to allow for the fiber formation. Similarly, neither Alg nor MAlg yielded successful results due to strong repulsive forces. Based on these results, PVA was selected as a support material to enable fiber fabrication with biopolymers. To achieve this, the electrospinning process was first optimized using PVA alone. A 12.8% *w*/*v* PVA solution was prepared by dissolving 6.5 g of PVA in 50 mL of Millie Q water at 70 °C under constant stirring for 2 h. The solution was then continuously stirred for an additional 24 h at room temperature to ensure complete dissolution and homogeneity. Subsequently, polymer blends were prepared to obtain fibers. The SEM image of PVA is shown in Figure S3A. To form fibers, sufficient positive interactions between the chains were necessary. In the case of PVA, these are mainly hydrogen bonds between hydroxyl groups, which need to extend in a continuous dimension (lengthwise). Figure S3A revealed continuous, uniform, and smooth fibers with an average diameter of 0.25 ± 0.08 µm. Due to the chain lengths and the high number of hydroxyl groups, the chains can easily extend lengthwise. Moreover, the flexibility of the chains enabled a homogeneous distribution of interactions, leading to uniform fiber morphology.

In parallel, solutions containing 3.2% Chi and Alg were prepared. For this purpose, Chi was dissolved in 1 M acetic acid and Alg was dissolved in MilliQ water, and both solutions were stirred for 24 h to ensure proper dissolution. Different volumes of the PVA and Chi or Alg solutions were mixed and stirred for 24 h to achieve various mass ratios between the two components. The viscosities of the PVA:biopolymer blends highlighted a different trend: as the proportion of Chi or Alg increased, so did the viscosity due to the higher interaction density of these compounds. However, polymer concentration had a more significant effect on viscosity than their ratio. As the concentration decreased, the number of interactions and overall viscosity were reduced due to dilution.

Additionally, Figure S3B presents the results for the PVA:Chi (2003 ± 110 cP) blend at an 8:2 ratio and 8% polymer concentration, where fibers with an average diameter of 0.19 ± 0.08 µm were obtained. These fibers showed significant diameter variations due to the stiffness of the chitosan chains, which affected the viscosity and limited homogeneity. However, with increased Chi concentration, fiber structure was lost, resulting in the formation of interconnected spheres with an average diameter of 0.41 ± 0.15 µm, along with areas lacking spherical structures. Due to the high viscosity of the solution (6492 ± 612 cP), droplets that were fully evaporated reached the collector, dissolving the spheres present in those regions. Subsequently, Figure S3C presents the PVA:MChi blend (1112 ± 49 cP) at the same 8:2 ratio and 8% concentration, where fibers with an average diameter of 0.14 ± 0.04 µm were obtained. Using PVA:MChi blend at a 6:4 ratio, fiber structures covered with spheres with an average diameter of 0.16 ± 0.05 µm were observed. Although fibers were formed, the 6:4 ratio was not considered desirable due to the large quantity of spheres produced. Compared to the fibers, these spheres had a lower surface-to-volume ratio, reducing the number of potential interaction sites. Similar observations can be made from the trials using both Chi and MChi. As the proportion of Chi increased, the formation of fibers became more challenging due to electrostatic repulsion. In general, the effect of PVA hydroxyl groups must be enhanced to achieve sufficient attractive interactions for fiber formation.

As observed, the diameter and distribution of MChi fibers were smaller compared to those of Chi (0.14 ± 0.04 µm vs. 0.19 ± 0.08 µm). This allowed for a higher quantity of fibers to be obtained with the same polymer mass as the dispersion was higher across each fiber. Moreover, the solvent-induced voids were absent in the methacrylated product, and the number of spheres was reduced, which facilitated fiber formation. Methacrylate groups hindered amine protonation and enabled the formation of weaker hydrogen bonds since the basicity of the amide group is lower than that of the amine group. At the same time, the number of hydrogen bonds that amides can form decreased due to the steric hindrance caused by the methacrylate groups. Therefore, forming a strong polycation became more difficult, reducing ion-ion interactions and allowing the chains to come closer.

Given that MChi fibers exhibited superior morphological characteristics and the possibility to perform crosslinking, it was decided to use PVA:MChi at an 8:2 ratio with an 8% polymer concentration and vary the flow rate. The SEM images of these experiments are shown in Figure S4 (see Supporting Information). At a flow rate of 0.2 mL·h^−1^, the fiber diameter was 0.20 ± 0.06 µm; at 0.35 mL·h^−1^, the diameter was 0.17 ± 0.05 µm; and at 0.45 mL·h^−1^, the diameter was 0.14 ± 0.04 µm. Increasing the flow rate reduced both the number of spheres and the fiber diameter while also decreasing diameter variation. As the flow rate increased, the amount of solution moving through the syringe per unit of time also increased, intensifying the interactions within the solution. This intensification of interactions had two effects on the MChi system: (I) it increased the electrostatic repulsion due to the polycationic nature and (II) enhanced the hydrogen bonding attraction produced by PVA’s hydroxyl groups. As a result, the fibers became thinner due to the increased repulsions, but their structure remained stable during the process because of the attractive forces. 

### 3.2. Electrospinning of Alginate-Based Fibers

Subsequently, Alg experiments were conducted. In Figure S5A, the SEM image for PVA:Alg (602 ± 35 cP) at an 8:2 ratio and 8% of polymer concentration is presented, showing fibers with a diameter of 0.22 ± 0.05 µm. In Figure S5B, the SEM image for PVA:MAlg (717 ± 32 cP) at same ratio and concentration is displayed, with fibers having a diameter of 0.18 ± 0.04 µm. As observed, the methacrylated product had a higher tendency to form spheres, even when using the same electrospinning parameters. 

No successful results were achieved with different PVA:MAlg ratios. Instead of observing slow deposition of a film, explosive droplets or highly unstable Taylor cones were formed [35,36].The polyanionic nature of Alg, where the electrostatic repulsions between the carboxyl groups of the chains have sufficient strength to overcome the hydrogen bonding attractions with PVA’s hydroxyls, needed a higher proportion of PVA to achieve fiber formation.

Finally, the fibers from the same PVA:MAlg blend system using different flow rates were analyzed. Using 0.25 mL·h^−1^ flow rate, fibers with a diameter of 0.21 ± 0.07 µm were obtained, while using 0.35 mL·h^−1^ flow rate, the diameter of fibers diminished to 0.18 ± 0.04 µm (Figure S6 in Supporting Information). In this case, results similar to those obtained with Chi were observed. As the flow rate increased, both the distribution of fiber diameters and the quantity of spheres decreased, while the fiber density increased. All of this occurred as the amount of polymer increased over time with increased flow, making more chains travel to the syringe tip per second, thus enhancing the number of interactions.

### 3.3. The Effect of Crosslinking on Electrospun Chitosan Fibers

Once fiber formation was achieved and optimized for each system and taking into account PVA’s high solubility in water, which could otherwise lead to fiber dissolution, crosslinking between fibers was performed to maintain structural integrity during water immersion. With the intended application of the membranes in mind, two different thermal initiators were used: AIBN (methanol-soluble) and V50 (water-soluble). For this purpose, only PVA with methacrylated biopolymers was analyzed as the methacrylate groups enable crosslinking, unlike the unmodified samples. 

For this purpose, PVA:methacrylated biopolymer 8:2 8% solutions were treated with 5% by mass of AIBN and/or V50 related to the polymer mass to maintain a 1:2 initiator-to-methacrylate group molar ratio. In the case of AIBN, it was dissolved in a negligible volume of methanol, comprising less than 5% of the solution’s total volume. On the other hand, V50, being water-soluble, was directly dissolved with the polymers. The resulting membranes were placed in an oven to complete the reaction of the initiators: at 115 °C for AIBN and 65 °C for V50, over a period of 24 h. Additionally, crosslinking with GA was performed by exposing the thermally treated samples to the vapors of 6 mL of an aqueous GA solution for 7 days. 

**Figure 1:**
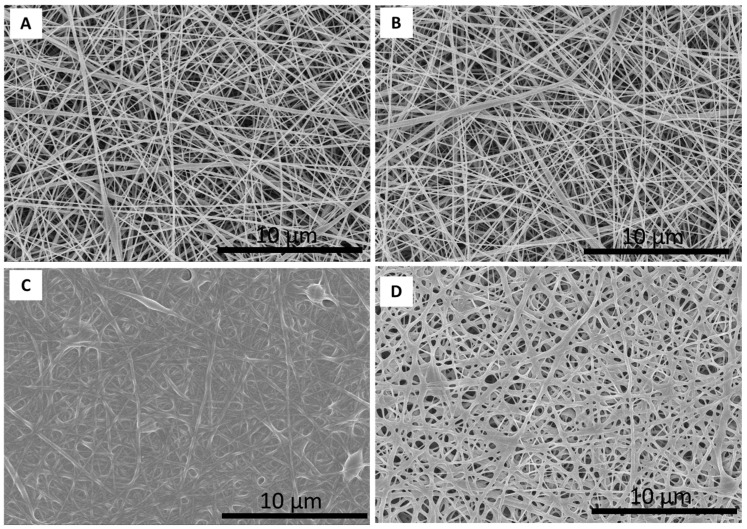
SEM images of PVA:MChi 8:2 8% electrospinning results after crosslinking with: (**A**) AIBN, (**B**) V50, (**C**) GA vapor, and (**D**) dual crosslinking using V50 and GA, at 5000× magnifications.

In Figure 1, the crosslinked systems are observed. Adding AIBN to the PVA:MChi system and heating for crosslinking at 115 °C resulted in fibers with a diameter of 0.14 ± 0.03 µm (Figure 1A). V50 was added and after being subjected to a thermal treatment at 65 °C for 24 h, fibers with a diameter of 0.15 ± 0.05 µm were obtained (Figure 1B). As observed, the fiber diameter increased following thermal crosslinking. This can be explained by the structural rearrangement required by the chains. Since the radicals generated in the methacrylate groups have only one reaction site, they needed to interact with the double bonds of other methacrylate groups. Due to heat diffusion and the torsional stress of the chains, the distance between the chains increased to facilitate crosslinking.

After analyzing the impact of thermal crosslinking, the effects of chemical crosslinking using GA and dual crosslinking were examined. For this purpose, the obtained PVA:MChi and PVA:MChi + V50 fibers were exposed to GA vapor for 7 days. Figure 1B displays the PVA:MChi 8:2 8% fibers mixed with V50 after thermal treatment. Conversely, Figure 1C shows the PVA:MChi 8:2 8% fibers after exposure to GA vapor, with the majority of the fiber structure degraded. This degradation occurred because GA reacted with hydroxyl and amino groups, promoting crosslinking between PVA-MChi, PVA-PVA, and MChi-MChi. Given GA’s gaseous state and high diffusion capability, crosslinking within and between fibers led to a loss of overall structural integrity. On the other hand, Figure 1D presents the fibers obtained via dual crosslinking (thermal treatment with V50 followed by GA vapor exposure) in PVA:MChi sample, resulting in fibers with a diameter of 0.27 ± 0.06 µm. It was evident that the fiber structure was maintained, alongside an increase in fiber diameter. The preservation of the structure, which did not occur with GA treatment alone, can be attributed to the initial thermal treatment. Through V50, the methacrylate groups reacted with each other, forming carbon–carbon bonds that restricted chain mobility, thereby limiting GA’s crosslinking effect to interactions between different fibers. The increase in diameter was due to crosslinking between adjacent fibers as GA promoted inter-fiber diffusion where methacrylate groups in MChi were not abundant.

### 3.4. The Effect of Crosslinking on Electrospun Alginate Fibers

**Figure 2:**
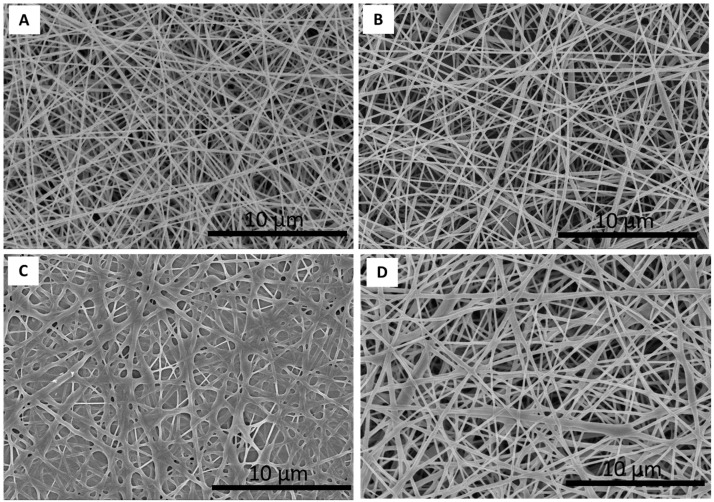
SEM images of PVA:MAlg 8:2 8% electrospinning results after crosslinking with: (**A**) AIBN, (**B**) V50, (**C**) GA vapor, and (**D**) dual crosslinking with V50 and GA, at 5000× magnifications.

Subsequently, the same process was applied to PVA:MAlg solutions. Figure 2A shows the fibers obtained by adding AIBN to a PVA:MAlg 8:2 8% solution and after being subjected to thermal treatment at 115 °C for 24 h, with fibers having a diameter of 0.165 ± 0.02 µm. In this case, the morphology of the fibers was notably irregular, with spheres and melted areas visible throughout. This irregularity was due to the methanol used to dissolve AIBN, which complicated fiber formation since Alg was insoluble in methanol. The fiber diameters obtained before and after thermal treatment with AIBN showed significant similarities in both MChi and MAlg systems, with an increase in diameter and a decrease in distribution. This outcome could be attributed to the effect of temperature on diffusion: since the glass transition temperature of PVA ranges between 50 and 80 °C, the mobility of polymer chains was enhanced, facilitating their diffusion and promoting radical crosslinking between methacrylate groups. This process increased the distance between chains along the fiber due to the steric hindrance caused by the methacrylate groups. In order to use a lower temperature to form radicals, V50 was added. Figure 2B shows the fibers obtained by adding V50 to a PVA:MAlg 8:2 8% solution, after being subjected to thermal treatment at 65 °C for 24 h with a resulting fiber diameters of 0.17 ± 0.02 µm. In this case, the theoretically expected increase in diameter was not achieved. Two different factors should be considered to explain this outcome. Firstly, a lower temperature was used for this crosslinking process compared to AIBN (65 °C vs. 115 °C), which reduced the energy available to the chains and limited their diffusion. Secondly, the initial fiber diameters before crosslinking were larger than in other cases, which may have required closer proximity between the chains to allow reactions between methacrylate groups, thereby reducing fiber diameter. On the other hand, GA vapor is used. Figure 2C displays the fibers after exposure to GA vapor for 7 days, where significant blending between fibers was observed. Similar to the PVA:MChi fibers, the reactivity of GA with hydroxyl groups enables PVA-MAlg, PVA-PVA, and MAlg-MAlg crosslinking, resulting in crosslinking throughout the entire length of the fibers. In the case of MAlg, less structural loss was observed compared to MChi due to the higher number of methacrylate groups in MAlg, which reduced the likelihood of crosslinking via GA. Finally, Figure 2D shows the fibers obtained through a dual crosslinking process (thermal treatment with V50 followed by GA vapor), where fibers with a diameter of 0.20 ± 0.05 µm were observed. In this case, the fiber structure is better preserved than in PVA:MChi system due to the higher degree of methacrylation in MAlg. This led to more crosslinking via V50, reducing diffusion and minimizing the impact of GA on fiber morphology. Adding GA with V50 slightly increased the diameter to 0.20 ± 0.05 µm, indicating that GA could contribute to thicker fibers due to crosslinking effects. GA alone did not yield measurable fiber diameters, likely due to insufficient fiber formation or unsuitable conditions for measurement. In summary, varying the biopolymer type, methacrylation, feed rate, and initiator type effectively controlled fiber diameter, enabling customization for specific applications.

### 3.5. The Effect of Water on Electrospun Membranes

After obtaining all different membranes and taking into account the aim of the study, the effect of water on the fibers was analyzed. For this purpose, the samples were placed in contact with Milli-Q water for 24 h. In all the PVA:MChi modified membranes when water contact extended to 24 h, the fibers were entirely lost. This phenomenon was attributed to the diffusion of the fibers. In fact, GA-based crosslinking did not provide significant rigidity, and the low methacrylation degree of MChi did not allow for high amount of crosslinking through V50. Consequently, the movement of the fibers induced by water was facilitated, leading to an easy loss of the overall structure.

**Figure 3:**
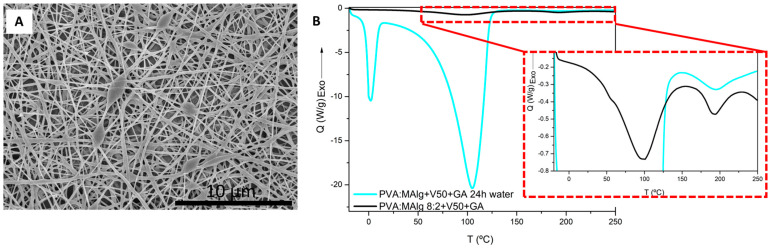
PVA:MAlg 8:2 8% electrospinning membrane after dual crosslinking with V50 and GA after 24 h exposure to water: (**A**) SEM image at 5000× magnification and (**B**) DSC thermograms of the same sample before being exposed to water (black) and after 24 h exposure to water (blue). Dotted lines show an enlargement of the signaled area.

The same procedure was followed for all PVA:MAlg membranes. Figure 3A displays the fibers obtained through dual crosslinking (V50 and GA) after being exposed to water for 24 h. As observed, the fiber structure was preserved due to the increased rigidity provided by the dual crosslinking process. The high degree of methacrylation resulted in a significant amount of crosslinking through V50, which reinforced the fiber structure by creating crosslinking points between the fibers. Additionally, GA also formed crosslinking points between the fibers, further enhancing the structural rigidity. Moreover, the thermograms presented in Figure 3B, obtained through differential scanning calorimetry (DSC) analysis, revealed that the membrane successfully retained the incorporated PVA, which was otherwise water-soluble, even after being immersed in water for 24 h because the fusion temperature of PVA at 200 °C was preserved. This observation underscored the effectiveness of the crosslinking process combined with the electrospinning technique, demonstrating its strong potential for developing membranes suitable for water-related applications.

### 3.6. Inclusion of Biocharcoal

**Figure 4:**
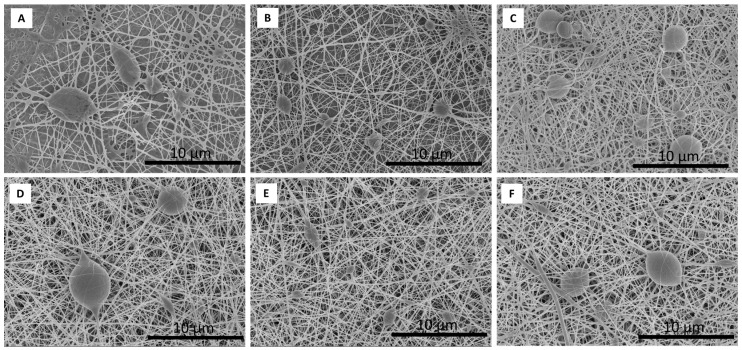
SEM images of PVA:MAlg 8:2 8% electrospun membranes: (**A**–**C**) using 1% biocharcoal and (**D**–**F**) using 2% biocharcoal. (**A**,**D**) without crosslinkers, (**B**,**E**) with V50 crosslinkers, and (**C**,**F**) with V50 and GA dual crosslinking. All images were obtained at 5000× magnification.

After optimizing fiber formation and making the decision of using PVA:MAlg system, which showed generally better results, it was decided to incorporate biocharcoal at a 1% and 2% ratio relative to the polymer mass. Although the precise structure of this material was not well defined, it was observed to contain aromatic groups, as well as hydroxyl and carboxylic acid groups. Due to these features, the introduction of this product was expected to enhance the adsorption of various pollutants owing to the newly introduced interactions [37]. Figure 4 shows PVA:MAlg 8:2 using biocharcoal as a filler. When incorporating 1% biochar without crosslinking, the biochar particles were clearly embedded within the fibers. However, upon crosslinking with V50, a more uniform distribution of fibers was observed, along with an increased presence of fibers extending across the entire particle surface. This effect was further amplified with dual crosslinking, leading to a significant enhancement in fiber distribution and density. With 2% biochar, all images revealed an improvement in fiber interconnection and an increase in the number of electrospun fibers. Notably, fiber density was higher both in the absence of a crosslinker and when using V50. However, under dual crosslinking conditions, no further enhancement was observed compared to the 1% biochar sample, suggesting a saturation effect in fiber formation.

**Figure 5:**
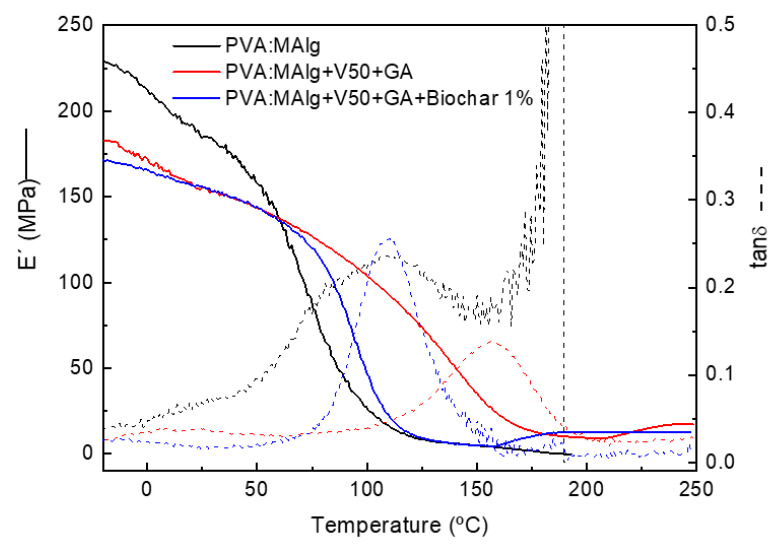
DMA analysis of Alg systems: without crosslinking (black), after dual crosslinking (red), and after the addition of biochar 1% and dual crosslinking (blue).

For this reason, it was decided to study the mechanical properties of different PVA:MAlg systems using dynamic mechanical analysis (DMA). DMA results presented in Figure 5 illustrate E′ and tan δ as a function of temperature for the alginate-based systems. The black curves correspond to crosslinker-free PVA:MAlg membrane, the red curves represent the sample subjected to dual crosslinking, and the blue curves show the performance of the sample containing 1% biocharcoal particles and subjected to dual crosslinking. All the samples exhibited a similar E′ of approximately 150–180 MPa at room temperature. The uncrosslinked sample had a slightly higher modulus, with a small decrease occurring when crosslinking the sample without or with biochar. In the case of the biochar addition, it was already reported [38] that the addition of biochar resulted in a decrease of E’. As the temperature increased, as seen in Figure 5, the E′ decreased due to the softening of the sample when passing from a glassy to a rubbery state. This caused an increase in tan δ at the peak of which the glass transition temperature (T_g_) can be determined. Thus, the T_g_ of the neat Alg material was approximately 110 °C, with a rather broad peak in tan δ, while the dual crosslinked samples (red curves) tan δ peak shifted to ~160 °C. However, the pristine membrane (black curves) underwent melting at higher temperatures (around 180–200 °C), possibly due to the high PVA content. This melting disappeared when the sample was crosslinked, not only improving its thermal resistance but also, as already mentioned, making it insoluble in water.

The incorporation of 1% biochar (blue curves) into the Alg matrix did not significantly affect E′ but caused a decrease in T_g_ (110 °C). This decrease may be due to the incorporation of biochar affecting the crosslinking degree of the polymer. However, PVA melting was not observed due to the crosslinking effect. Comparing these systems, it was evident that dual crosslinking consistently enhanced the mechanical performance of the Alg matrix. These results highlighted the dual crosslinking method as the superior strategy for achieving high mechanical strength and thermal stability in biopolymer systems. Biocharcoal, while effective as a reinforcing filler, may be more beneficial when combined with crosslinking techniques to maximize its potential.

**Figure 6:**
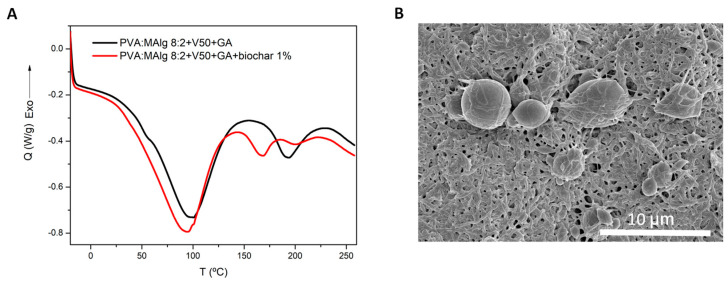
Analysis of PVA:MAlg 8:2 after dual crosslinked samples: (**A**) DSC thermograms (without and with biochar 1%) and (**B**) the SEM image of the sample with 1% biocharcoal after exposure to water for 48 h.

Additionally, DSC analysis was performed on the crosslinked samples with biochar addition (1%) to verify the effective incorporation of inorganic particles into the membranes (Figure 6A). The appearance of new melting peaks was observed, likely due to interactions between the fibers and the biochar, demonstrating that the fillers were effectively integrated into the matrix. Furthermore, SEM analysis of the biochar-containing samples exposed to water for 48 h (Figure 6B) revealed partial degradation of the fibers, although their presence remained noticeably evident. This effect could be attributed to biocharcoal: as the fibers formed around the particles, the diffusion of the polymer chains was hindered, reducing the efficiency of the crosslinking process and diminishing the water resistance that would otherwise be achieved in the fiber structure.

The first transition observed in both cases was due to the evaporation of water molecules trapped in the structure. The subsequent distinguishing transition was the melting temperature of PVA, which occurred at 193 ± 1 °C in the PVA:MAlg 8:2 system without biocharcoal. This temperature was lower than the typical melting temperature of PVA with an 88% hydrolysis level, which was 220 °C [39]. This phenomenon occurred due to the interaction with Alg where the PVA-MAlg interactions weakened the PVA-PVA interactions, thus weakening the crystalline structure [40]. Concurrently, the incorporation of methacrylate groups introduced steric hindrances during the formation of crystalline regions, further compromising the structure. In the case of the PVA:MAlg 8:2 with 1% biocharcoal, the melting temperature of PVA was observed in two stages. Initially, most of the crystalline structure was lost at 168 ± 1 °C, with subsequent melting occurring at 200 ± 1 °C. This can be attributed to the influence of biocharcoal particles, which acted as nucleation points in the fibers, transforming and weakening the crystalline structure that polymer chains could have, leading to melting at 168 ± 1 °C [38]. 

Additionally, both samples (PVA:MAlg 8:2 without biocharcol and PVA:MAlg 8:2 with biocharcoal 1%) showed similar thermal degradation profiles in thermogravimetric analysis (TGA) and the derivative of weight loss (DTG) curves with slight differences in peak intensities and exact temperatures (Figure S7 in Supporting Information). This could indicate that while they share comparable components, minor compositional or structural differences affected their thermal stability. In fact, both samples underwent multiple stages of decomposition, with primary degradation occurring in the 200–400 °C range, a secondary stage near 450–500 °C, and a stable residue forming after 500 °C. Moreover, an initial weight loss at lower temperatures was observed in both samples, which was commonly attributed to the loss of absorbed water or volatile compounds. This was observed as a minor peak in the DTG curve around 100–150 °C in both plots. This was an expected behavior in materials containing PVA and Alg, which were hydrophilic and retained some water in their structure. The main degradation was around 200–400 °C for both samples. These peaks corresponded to significant mass losses, likely due to the breakdown of the main polymeric structure. The main degradation of PVA and Alg occurred at this temperature as both structures decomposed at moderate temperatures [41]. The presence of peaks in this range in both samples suggested the breaking of the PVA and Alg chains. 

Additional DTG peaks were observed in both samples after the primary decomposition, around 450–500 °C. This could indicate the degradation of remaining high-stability fractions or more crosslinkedstructures. The sample with biochar exhibited a second peak in the region of 450–500 °C, which could indicate higher thermal stability or an additional phase of degradation. Biochar is known for its high thermal stability, and its inclusion can delay or modify the decomposition of the material, acting as a barrier and possibly increasing the amount of residue at the end of the analysis. In addition, biochar could facilitate the formation of carbonized structures that were more resistant to high temperatures. Both samples reached a plateau after 500 °C, suggesting the formation of a stable residue. The biochar sample likely had a higher residue at the end of the test (after 500 °C), which was consistent with its presence, as it is a stable form of carbon that cannot decompose easily. This elevated residue was consistent with the expected thermal behavior of materials with strong carbon content.

### 3.7. Adsorption of Methylene Blue

**Figure 7:**
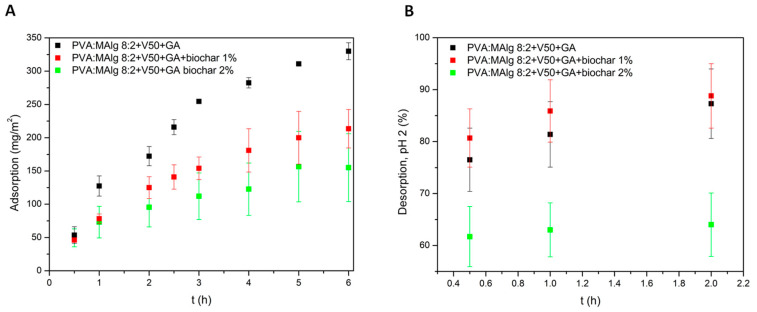
(**A**) MB adsorption and (**B**) MB desorption curves at pH 2 of three different systems: (i) PVA:MAlg 8:2 + V50 + GA (black squares), (ii) PVA:MAlg 8:2 + V50 + GA + biochar 1% (red squares), and (iii) PVA:MAlg 8:2 + V50 + GA + biochar 2% (green squares).

Given the intended application of these membranes for the removal of water pollutants, a crucial step in their evaluation involved assessing their adsorption and desorption capacities. After a comprehensive characterization of their morphology using SEM analysis, along with their mechanical and thermal properties, the next phase of this study focused on evaluating their ability to adsorb MB, a model contaminant commonly used in water treatment research. Subsequently, desorption experiments were conducted to determine the extent to which the membranes can be regenerated and reused. These findings provided essential insights into the feasibility of employing these electrospun biopolymer-based membranes as efficient and sustainable adsorbents for wastewater remediation. MB adsorption performance of the developed materials over time is depicted in Figure 7A, comparing three different systems: (i) PVA:MAlg 8:2 + V50 + GA (black), (ii) PVA:MAlg 8:2 + V50 + GA + biochar 1% (red), and (iii) PVA:MAlg 8:2 + V50 + GA + biochar 2% (green). The adsorption capacity (mg/m^2^) was monitored for up to 6 h, providing insights into the influence of biochar content on the adsorption efficiency.

During the initial adsorption phase (t = 1 h), the biochar free sample (black) exhibited an adsorption capacity of approximately 125 mg/m^2^, significantly outperforming the biochar-containing samples, which showed values around 75 mg/m^2^ for both 1% and 2% biochar loadings, respectively. This trend suggested that the presence of biochar initially hinders adsorption, possibly due to changes in the surface area or active site availability caused by the filler. Over time, all systems showed an increase in adsorption capacity, with the sample without biochar maintaining the highest values throughout the experiment. After 3 h, the biochar-free sample reached 250 mg/m^2^, while the red (1% biochar) and green (2% biochar) samples exhibited lower adsorption capacities of approximately 150 mg/m^2^ and 100 mg/m^2^, respectively. The incorporation of biochar, particularly at 2%, consistently resulted in reduced adsorption performance. After 6 h, the baseline system achieved the highest adsorption capacity, peaking at ~320 mg/m^2^, whereas the 1% biochar sample reached ~200 mg/m^2^ and the 2% biochar sample only ~150 mg/m^2^. The plateau observed in all samples suggests that adsorption equilibrium was reached, with the filler-free material demonstrating superior performance.

The negative correlation between biochar content and adsorption capacity might be attributed to a reduction in the active sites or possible diffusion limitations within the matrix. While biochar is generally known for its high surface area, its integration into the PVA:MAlg matrix may alter the porosity or block functional groups necessary for effective adsorption. Additionally, higher biochar loadings (2%) did not translate to improved adsorption, reinforcing the notion that the interaction between biochar and the polymeric matrix may not be fully optimized for adsorption applications.

The results indicated that while dual crosslinking (V50 + GA) enhanced the adsorption capacity of the material, the inclusion of biochar, contrary to expectations, decreased performance. Future work could explore the optimization of biochar dispersion or surface modification strategies to enhance the synergy between the biochar and the polymer matrix, potentially unlocking higher adsorption efficiencies.

The desorption performance of MB at pH 2 over 2 h from the developed materials is shown in Figure 7B, comparing three systems: (i) PVA:MAlg 8:2 + V50 + GA (black), (ii) PVA:MAlg 8:2 + V50 + GA + biochar 1% (red), and (iii) PVA:MAlg 8:2 + V50 + GA + biochar 2% (green). The percentage of desorption capacity was monitored to evaluate the release efficiency of the previously adsorbed MB. The filler-free system exhibited a gradual increase in desorption capacity over time, reaching a maximum of 90% in 2 h, demonstrating its desorption. Alg component demonstrated a pH-dependent behavior due to its functional groups. Under acidic conditions, protonation of these groups occurred, weakening their interaction with MB as stronger interactions with the abundant H^+^ ions in the medium dominated. This shift in acidity reduced the affinity between Alg and MB, improving the desorption capacity of the system. The system with 1% biochar (red) showed similar desorption behavior. 

When contrasted with the adsorption performance from Figure 7A, the 1% biochar system balanced moderate adsorption with partial desorption, offering a compromise between capacity and release control. Meanwhile, the non-filler addition system showed both high adsorption at neutral pH and high desorption at pH 2, indicating a more dynamic but less retentive behavior. At acidic pH, MB was not released from the membrane containing biochar, indicating that biochar integration could be beneficial when desorption is not desired. This makes biochar-containing membranes suitable for short-term applications where the material is intended for single use and subsequent disposal. Conversely, if the goal is to reuse the membrane, the presence of biochar may not be advantageous. Overall, these findings highlight that the incorporation of biochar, particularly at higher concentrations, enhanced the binding strength of MB to the polymer matrix, effectively preventing desorption. This characteristic is particularly valuable in applications requiring strong dye capture and stability, such as in single-use filtration systems or scenarios with a low risk of contamination. However, for applications where controlled desorption is necessary, biochar-free systems would provide a more effective solution.

## 4. Conclusions

This study highlights the potential of electrospinning as a robust technique for producing nanofibers with controlled morphology and tailored properties. By optimizing polymer/copolymer/solvent systems—including PVA, Chi, Alg, and PVA:biopolymer mixtures—this work demonstrates how viscosity and polymer concentration critically influence fiber formation. Methacrylation using methacrylic anhydride proved particularly effective, with MAlg achieving a higher degree of modification than MChi, leading to improved crosslinking efficiency and enhanced fiber morphology.

The crosslinking effect has proven to be a critical factor throughout this study, demonstrating the substantial differences observed in the obtained membranes. The most effective crosslinking for both biopolymer systems was achieved through the combination of the thermal initiator V50 and GA vapor, showing a clear correlation with the degree of methacrylation obtained. In the case of the Chi system, due to its lower degree of methacrylation, the membrane structure could not be maintained after 24 h of water immersion. Conversely, the Alg system preserved fiber integrity even after 48 h, highlighting its potential for applications in water treatment and purification processes.

The study also revealed that while biochar particles integrate well within the polymer matrix and contribute to enhanced mechanical properties, their impact on adsorption—specifically for MB—was not as promising. The addition of biochar did not improve adsorption performance and showed limited benefits for desorption, suggesting that its use may be advantageous if the reuse of membranes is not required. Future studies could explore whether similar outcomes occur with other contaminants, broadening the understanding of biochar’s role in adsorption systems.

Future research should focus on optimizing biopolymer content and exploring alternative modifications. Although total removal or chemical consumption of crosslinking agents is assumed, trace amounts of these could be trapped in the polymeric structure and be freed into the aqueous environment its designed to remediate. If used agents are not environmentally friendly, this event would weaken the advantages of the membrane due to its own polluting action, hence the need for further research on development of greener crosslinking strategies to enhance material performance. Investigating new polymers and multilayer nanofiber structures could further expand their potential for water treatment and other applications.

## Data Availability

The original contributions presented in this study are included in the article/supplementary material. Further inquiries can be directed to the corresponding author(s).

## Supplementary Materials

The following supporting information can be downloaded at: https://www.mdpi.com/article/10.3390/polym17070988/s1, Figure S1: ^1^H-RMN spectrum of methacrylated chitosan (MChi); Figure S2: ^1^H-RMN spectrum of methacrylated alginate (MAlg); Figure S3: SEM images of electrospinning obtained samples for PVA and biopolymer blend at an 8:2 ratio with an 8% polymer concentration. (A) PVA, (B) PVA:Chi, and (C) PVA:MChi, captured at 5000× magnification; Figure S4: SEM images of electrospinning results for PVA:MChi at an 8:2 ratio with an 8% polymer concentration using different flow rate: (A) 0.2 mL·h^−1^, (B) 0.35 mL·h^−1^, and (C) 0.45 mL·h^−1^, at 5000× magnifications; Figure S5: SEM images obtained using the electrospinning technique for PVA:biopolymer blends at an 8:2 ratio with an 8% polymer concentration. (A) PVA:Alg and (B) PVA:MAlg, at 5000× magnifications; Figure S6: SEM images of results from PVA:MAlg at an 8:2 ratio with an 8% polymer concentration and different flow rates: (A) 0.25 mL·h^−1^, and (B) 0.35 mL·h^−1^, at 5000× magnifications; Figure S7: TGA and DTG analysis for (A) PVA:MAlg 8:2 8% sample after V50 and GA treatment and (B) same sample adding 1% biocharcoal.

## Abbreviations

AIBN	azobisisobutyronitrile
Alg	Alginate
Chi	Chitosan
DMA	dynamic mechanical analysis
DSC	Differential scanning calorimetry
E′	Storage modulus
E′′	Loss modulus
GA	Glutaraldehyde
MAlg	Methacrylated alginate
MB	Methylene blue
MChi	Methacrylated chitosan
PVA	Polyvinyl alcohol
SEM	Scanning electron microscopy
Tan δ	Loss factor or E′′/E′
T_g_	glass transition temperature
TGA	Thermo-gravimetric analysis
V50	2,2’-azobis(2-amidinopropane)dihydrochloride
