# Supplementary material for: The Importance of Crosslinking in Electrospun Membranes for Water Contaminant Removal
Source: Polymers (Basel). 2025 Apr 5;17(7):988. doi: 10.3390/polym17070988 (PMC11991501; doi:10.3390/polym17070988)
Supplement: Supplementary file 1 [file polymers-17-00988-s001.zip › polymers-3544405-supplementary.pdf]

Supporting information

The Importance of Crosslinking in Electrospun Biomembranes for Water Contaminant Removal

Peio Martinez-Goikoetxea[1], José Manuel Laza[1], Julia Sanchez-Bodon[1], José Luis Vilas-Vilela[1,2], and Antonio Veloso-Fernández[1,*]

1 Grupo de Química Macromolecular (iMacroMat), Departamento de Química Física, Facultad de Ciencia y Tecnología, Universidad del País Vasco UPV/EHU, 48940, Leioa, Spain

2 BCMaterials, Basque Center for Materials, Applications and Nanostructures, UPV/EHU Science Park, 48940 Leioa, Spain

*Correspondence: antonio.veloso@ehu.eus; Tel.: +34-946-01-5965

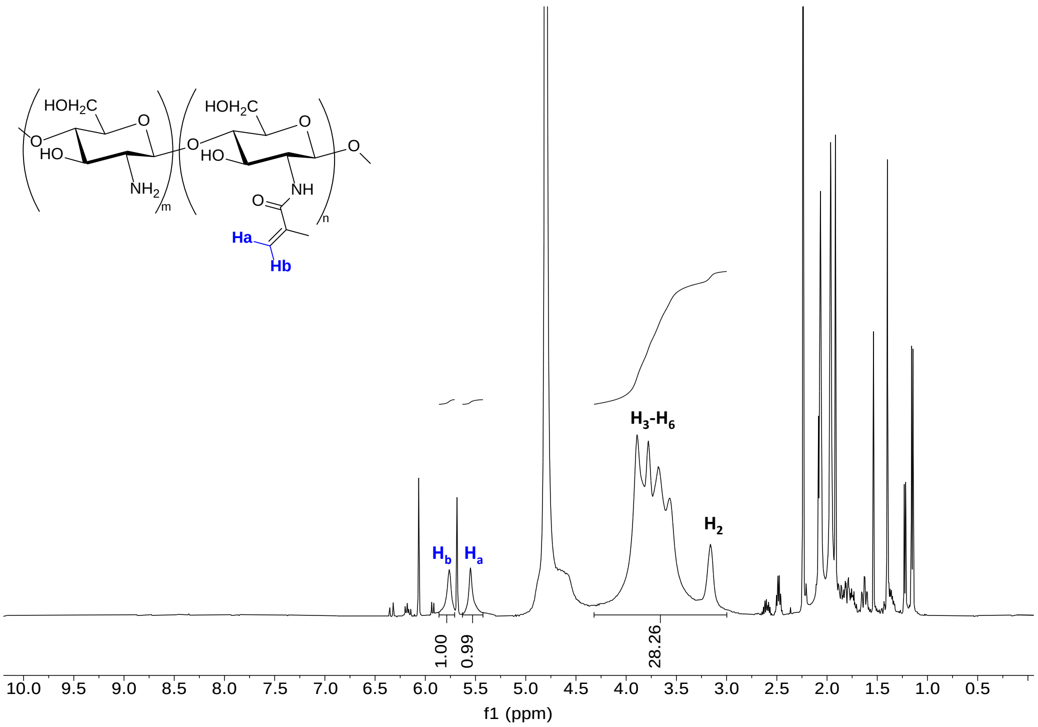

Figure S1. **^{1}H-**RMN spectrum of methacrylated chitosan (MChi)

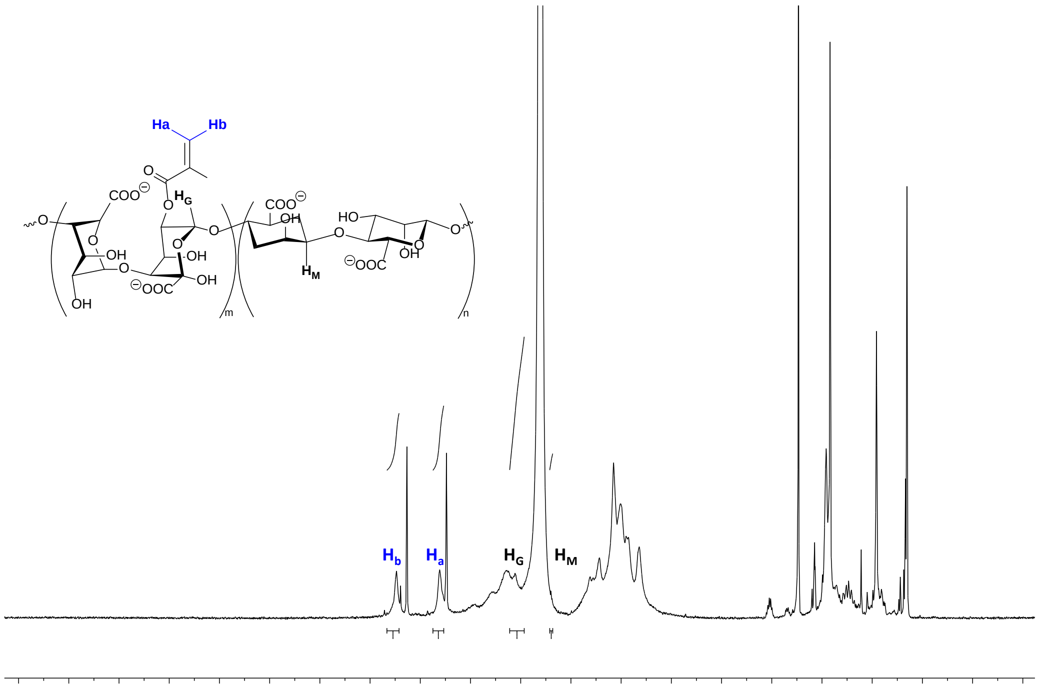

Figure S2. **^{1}H-**RMN spectrum of methacrylated alginate (MAlg).

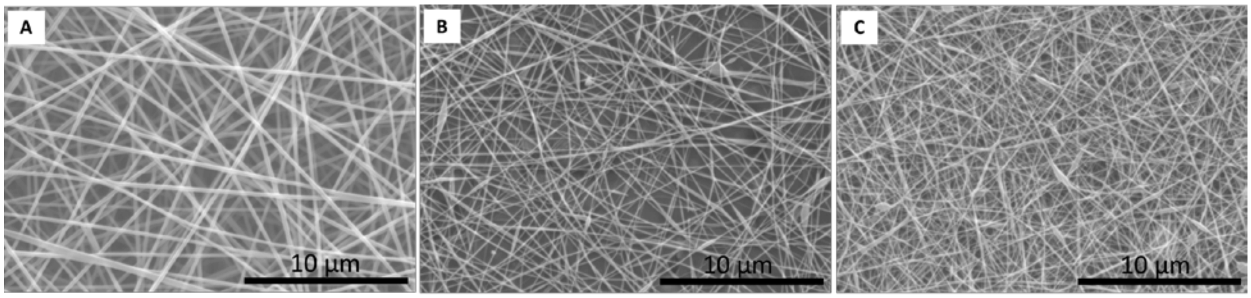

Figure S3. SEM images of electrospinning obtained samples for PVA and biopolymer blend at an 8:2 ratio with an 8% polymer concentration. A) PVA, B) PVA:Chi, and C) PVA:MChi, captured at 5000x magnification.

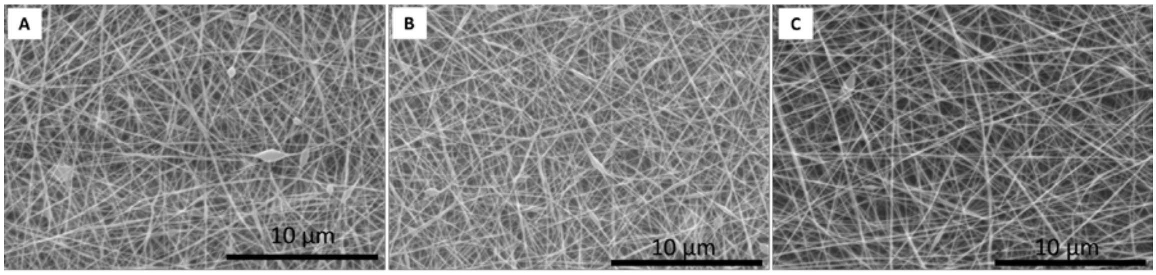

Figure S4. SEM images of electrospinning results for PVA:MChi at an 8:2 ratio with an 8% polymer concentration using different flow rate: A) 0.2 mL·h^{-1}, B) 0.35 mL·h^{-1}, and C) 0.45 mL·h^{-1}, at 5000X magnifications.

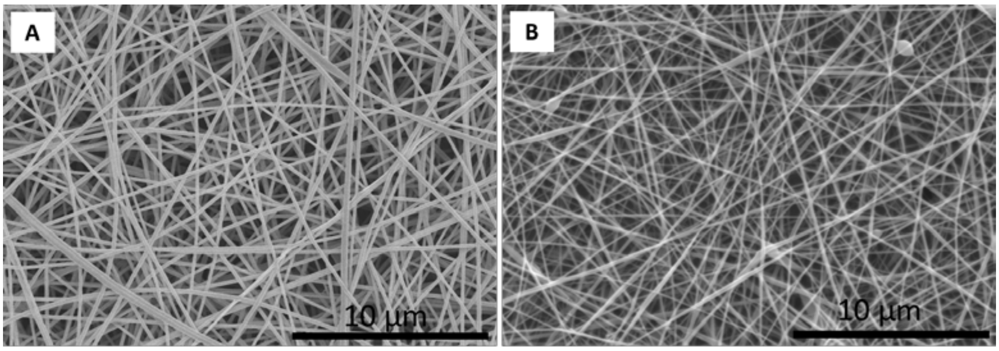

Figure S5. SEM images obtained using the electrospinning technique for PVA:biopolymer blends at an 8:2 ratio with an 8% polymer concentration. A) PVA:Alg and B) PVA:MAlg, at 5000x magnifications.

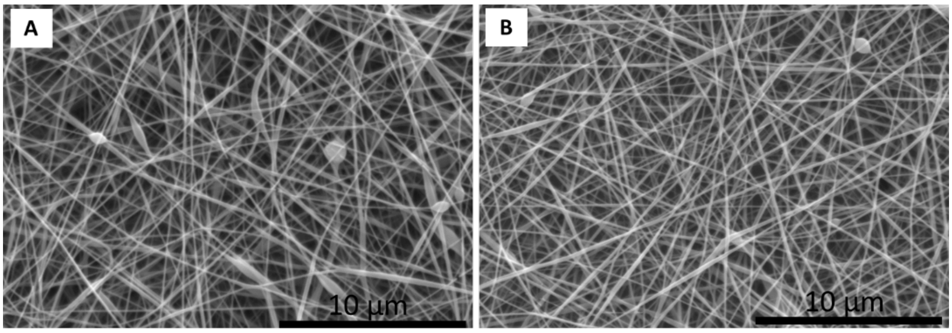

Figure S6. SEM images of results from PVA:MAlg at an 8:2 ratio with an 8% polymer concentration and different flow rates: A) 0.25 mL·h^{-1}, and B) 0.35 mL·h^{-1}, at 5000x magnifications.

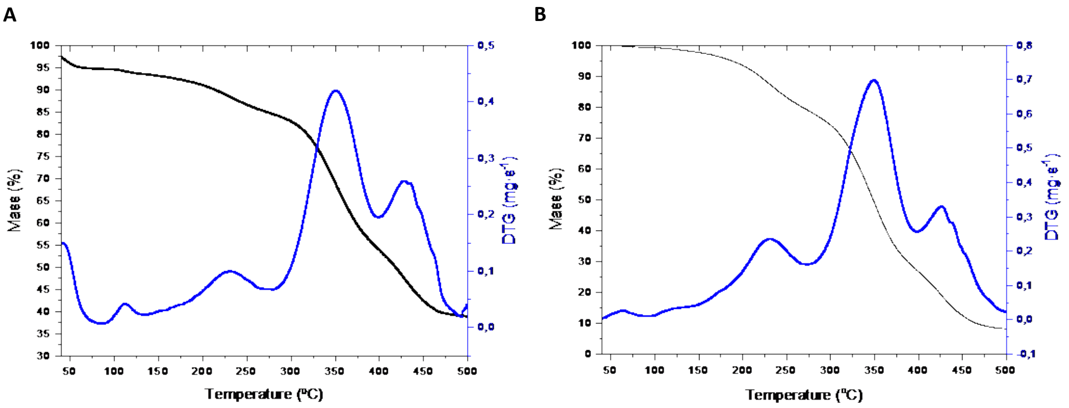

Figure S7. TGA and DTG analysis for A) PVA:MAlg 8:2 8% sample after V50 and GA treatment and B) same sample adding 1% biocharcoal.
